# Are All the Races of Men Descended from a Single Pair?

**Published:** 1881-05

**Authors:** Harvey L. Byrd

**Affiliations:** Professor of Obstetrics in Washington University of Baltimore


### POPULAR SCIENCE DEPARTMENT.

### ARE ALL THE RACES OF MEN DESCENDED FROM A SINGLE PAIR?

BY HARVEY L. BYRD, M. D.,
Professor of Obstetrics in Washington University of Baltimore.

An affirmative answer to the above inquiry would, perhaps, afford consolation, and might prove satisfactory even, to certain fanatical individuals of the white race, who believe, or at least profess to believe, that the black skin, woolly hair and thick lips of the African place him a degree or two higher in the scale of the *genus homo* than that occupied by themselves; and, probably, certain other well-meaning but timid persons might be inclined to suspect that we do not venerate religious teaching, or the opening paragraph of the "Declaration of Independence," as they do, should we venture to answer the proposition in the negative. *The Baltimore Medical Journal* is an appropriate medium through which to present to the profession such facts and intelligence of a scientific character as relate to the physical peculiarities of the human race, and the times require that these developments should be recorded and permitted to flow *pari passu* with new discoveries in pathology and therapeutics. With the political aspect of the question, and the absurd ideas and preposterous conclusions of fanatics, we can here have nothing to do; and, however much we may deplore the wickedness or commiserate the blindness of those who attempt the subversion of the immutable laws of Jehovah, in order to the carrying into practical operation their ideas and conclusions, the pages of a medical journal cannot be prostituted to their refutation.

We have abundant reason for believing that the vast "chain of being which from God began" was formed upon a plan so unerring in its character that it cannot now be changed in the smallest essential element or particular. The design and harmony of action which we so clearly and unmistakably see exhibited in all departments of nature's works that have been explored and comprehended by man, assures us we are not mistaken in concluding that order, symmetry and harmony had their birth in the early dawn of creation, ere the "morning stars sang together" at beholding the newly formed world. In the examination and study of this great question, we should, for obvious reasons, give so much attention to the formation of the earth as to discover its perfect adaptation to the support of vegetation before an herb or tree was planted upon its prolific and generous bosom. At proper and regular periods, in fulfilment of the great plan of the Architect of universal nature, animal life sprang into existence, each *variety* in its own peculiar form and proper time, in obedience to laws which the Creator determined should govern them and their successors, so long as their existence might be continued on the earth. Reasoning thus from facts as well as from analogy, we are forced to the conclusion that *all* living beings—certainly *all distinct* species—have continued to increase and multiply from the time of their creation, in strict conformity and obedience to the laws which were ordained for their government and development. The fact that nature can never be otherwise than true to herself is apparent on every page of her vast and glorious volume; and the assertion is constantly verified by physicists and astronomers, in their investigations, that "science is but nature understood." Naturalists, of whom comparative anatomists form no unimportant portion, are rapidly advancing in the knowledge of minutiæ of their departments; and if we are permitted to judge from such facts as have been developed within a few years, we are led to the conclusion that the next decade will find the latter occupying as safe and satisfactory ground as that which qualitative analysis secures to the chemist.

Success in any of the natural or physical sciences is attainable only through a knowledge of the laws relating thereto, however much we may be captivated by beautiful and fanciful theories. In natural history many and important facts have been discovered, analyzed and systematized. Thus we have a satisfactory standard for the vertebrata. It has been shown that a whole type of vertebrata is built upon a distinct plan. Professor Owen has demonstrated this and kindred facts in England; the same have been rendered familiar to the naturalists of France by the illustrious Geffroy St. Hilaire, and Professor Agassiz is adding greatly to this knowledge on this continent. These and other discoveries prove conclusively that there is no foundation whatever for the theory that "animals have been successively changed so as to form the great variety which we see." The vertebrata, as above stated, are built upon an entirely different and distinct plan from any other class or division of animals, and, therefore, have but little in common with them. The plan of the articulates is also well understood, and they differ as widely from the vertebrates as either does from the mollusks, or the latter from the radiates. These facts incontestibly show the utter *impossibility* of a transmutation of animals. The foregoing discoveries have been adduced to prove that a *law once inaugurated ever after* continues as unchangably its influence in the *animate* as in the *inanimate* works of creation. What is true of the laws governing the forms and habits of animals must be equally true of those regulating their modes of propagation. A law, therefore, which is now known to *prohibit* the breeding *inter se* of a limited number of animals of the same type for any considerable length of time, must have existed commensurate with its creation! The fact is as well known to the uneducated husbandman as to the scientific physiologist that many of our domestic animals, such as horses, cows, sheep and hogs, *degenerate* and ultimately *die out* by long breeding in and in, and that fresh blood must be introduced—even from a neighboring pasture—in order to the perpetuation of the original status of the stock. I am informed that our domestic poultry affords additional proof of the same fact, and that it is necessary to change the breeds occasionally in order to increase or even preserve the original value of "the denizens" of the barn-yard. The foregoing facts, doubtless, apply with equal force to *all* animals. Examples, fortunately, are not seen at the present day of the interbreeding of brothers and sisters, or parents and children, in the *genus homo*, as occurs in the lower domestic animals; but we witness, in the imbecile and degenerate offspring of near relatives who have intermarried for several generations, unmistakable evidence of violated natural law.

Whole families have become extinct from this interbreeding. Such being the known results of violated law in the present age, it is surely not unreasonable to infer that the same would have proven true of similar interbreedings at any previous period of time.

We are hence led to the conclusion that it is *physiologically certain* that neither man nor the lower animals could have been perpetuated from a *few*, much less from a *single* pair. Nature has unmistakably decreed *several* species or types of the same genus among the lower orders of animals, and careful examination and comparison force the conclusion on our mind that at least the *three* well-defined and prominently distinguishable types of men, viz.: the Caucasian, Mongolian and Negro races, have continued to present, from the periods of their creation, the same well-defined differences and characteristics which so eminently distinguish them now. Their anatomical, physiological, mental and moral characteristics are too well known to intelligent physicians to require a notice of them here. The idea of time and climate having wrought out these distinctions in color, *et cetera*, as was asserted by a few ethnologists of eminence some years ago, may be still entertained by prejudiced persons; but it is apprehended that no naturalist of respectable attainments in the anatomy, even of the types above mentioned, would now venture to assert that they were *all* cast *originally* in the same form and color, or that these three races of men are from common stock, much less the descendants of a *single pair*. All experience teaches that nature is and must continue true to her own great laws. Our professional success depends entirely upon the use we make of her laws in the treatment of disease, as certainly, though we may not always appreciate them so clearly, as the laws of mathematics or mechanics in the solution of problems or the construction of edifices and machinery.

The timidity with which this great question is approached by some good men is incomprehensible on any other ground than *fear*, lest scientific investigations might prove *too much*, and thereby tend to unsteady their construction or understanding of the Mosaic account of the origin of man. Such apprehensions are natural enough, until the important truth is learned that the Bible was never intended as a teacher of science. A limited acquaintance only with anthropology exposes the diversity of types, and convinces us that the races must have sprang from different sources, while a fuller comprehension of the science certainly establishes man as the highest, as he is the most perfect work of Nature. Created by an intelligence so vastly superior to his own, that each step in the study of his physical and phychological composition reveals in unmistakable language that he was mainly formed for immortality. Such being the case, the Bible—the revealed will of God—and science must, in the very nature of things, completely harmonize in all their precepts and teachings, when the former speaks at all.

The demands of humanity and of the age in which we live require at the hands of our profession a clear and full expression of the revelations of science on this vitally important subject. To us, intelligent religious minds appeal for light amid the dogmas of "fallible" men.

On a subsequent occasion we purpose inviting the attention of your readers to some practical facts as to the liability of the Caucasion and African races to particular diseases, and the manner and extent to which they are respectively impressed by certain morbific and therapeutic agents, as interesting professional *proof of their different sources of origin.*

NOTE.—The foregoing article is republished from the *Baltimore Medical Journal* of 1870, at the request of several of our subscribers.

